# Hierarchically Porous Microspheres by Thiol-ene Photopolymerization of High Internal Phase Emulsions-in-Water Colloidal Systems

**DOI:** 10.3390/polym13193366

**Published:** 2021-09-30

**Authors:** Stanko Kramer, Peter Krajnc

**Affiliations:** PolyOrgLab, Faculty of Chemistry and Chemical Engineering, University of Maribor, Smetanova 17, 2000 Maribor, Slovenia; stanko.kramer@um.si

**Keywords:** polyHIPE, porous microspheres, thiol-ene polymerization, suspension polymerization, multiple emulsions, adsorption, methylene blue, porous polymers

## Abstract

A facile method for the preparation of hierarchically porous spherical particles using high internal phase water-in-oil-in-water (w/o/w) double emulsions via the photopolymerization of the water-in-oil high internal phase emulsion (w/o HIPE) was developed. Visible-light photopolymerization was used for the synthesis of microspherical particles. The HIP emulsion had an internal phase volume of 80% and an oil phase containing either thiol pentaerythritol tetrakis(3-mercaptopropionate) (PETMP) or trimethylolpropane tris(3-mercaptopropionate) (TMPTMP) and acrylate trimethylolpropane triacrylate (TMPTA). This enabled the preparation of microspheres with an open porous morphology, on both the surface and within the microsphere, with high yields in a batch manner. The effect of the thiol-to-acrylate ratio on the microsphere diameter, pore and window diameter, and degradation was investigated. It is shown that thiol has a minor effect on the microsphere and pore diameter, while the acrylate ratio affects the degradation speed, which decreases with increasing acrylate content. The possibility of free thiol group functionalization was demonstrated by a reaction with allylamine, while the microsphere adsorption capabilities were tested by the adsorption of methylene blue.

## 1. Introduction

Porous polymers are an increasingly researched class of materials due to their specific characteristics and wide applicability. They can be used as flow-through supports [1], in oil spill clean-ups [2], in extractions [3], in catalyses [4], as adsorbents and absorbents [5], as shape-memory materials [6], in heavy metal removal [7], in separation processes (filters) [8], as controlled-release matrices [9], wound dressings [10], tissue engineering [11], advanced healthcare applications [12], etc. Furthermore, easy and relatively cheap processing and the possibility of controlled degradation make porous polymers an alternative to porous inorganic materials. One way to prepare porous polymers is by using high internal phase emulsion (HIPE) templating. HIPEs are emulsions that comprise an internal phase with a volume fraction of at least 74% and a continuous phase into which the internal phase is dispersed. In HIPE templating, the continuous phase is polymerized and forms an interconnected material known as polyHIPE. PolyHIPEs are mostly formed from water-in-oil (w/o) HIPEs; however, examples of oil-in-water (o/w) are also known [13]. PolyHIPE materials possess a unique interconnected cellular porous macroscopic structure, which is the result of the formation of the precursor high internal phase emulsion. In such emulsions, droplets of the internal phase are packed in the closest possible proximity, while the surfactant molecules prevent both phase separation and phase inversion. In order to achieve kinetical stability of the high internal phase emulsion, which is necessary for retaining the unique morphology during the polymerization, the polarity of the aqueous phase is usually increased by the addition of salts. When the polymerization of the continuous phase is initiated, and the viscosity and the density of the continuous phase increase, resulting in areas of high pressure at the thinnest points of droplet contact. This results in polymer film rupture and the formation of the unique polyHIPE morphology, where primary pores (usually termed cavities or voids) are the result of the emulsion internal phase droplets, while the interconnecting pores (usually termed windows) are the result of the density changes during the polymerization. The surfactant(s) and salt(s) are effective components in the prevention of phase separation (interfacial tension) and Ostwald ripening, which causes significant droplet size dispersion. The formed polyHIPEs are generally molded into the form of the vessel in which they polymerize. As the shape of the polyHIPEs can impact their application, control of the produced shape (microspheres, rods, fibers, and membranes) has become an important field of research. Differently shaped polyHIPEs (especially microspheres) can be produced by using multiple emulsions [14], frozen polymerization [15], precipitation polymerization [16], phase inversion [17], sedimentation polymerization [18], and microfluidic fabrication [19]. The methods most widely used to produce porous microspheres are microfluidic fabrication followed by suspension polymerization. Microfluidics is a more complex and advanced version of suspension polymerization [20]. In microfluidics, the droplets for microsphere formation are generated one-by-one in a controlled and reproducible manner, which results in a narrow size distribution of the synthesized microspheres. In comparison, suspension polymerization produces a wider size distribution of the microspheres, as the polymerizable phase is mixed with the suspension phase, which results in the formation of droplets of different sizes [21].

The production of polyHIPE microspheres using suspension polymerization was first mentioned in the form of a patent developed by Li et al. [22]. Desforges et al. [14] introduced vinylbenzyl chloride (VBC) into styrene-divinylbenzene (DVB) polyHIPE microspheres to enable further functionalization of the prepared particles, whereas our group prepared VBC–DVB polyHIPE microspheres and functionalized them with different functionalities [23]. In addition to the styrene-based polyHIPE microspheres, the synthesis of acrylate-based polyHIPE microspheres was also demonstrated [24]. The spherical structure of the materials and their chemical variety made them particularly interesting as materials for wastewater treatment [16,25,26]. Zhu et al. [16] used precipitation polymerization of Pickering-type emulsions to prepare millimeter-sized beads using hydroxyl propyl cellulose and acrylic acid for the removal of Cu^2+^ and Cd^2+^. In comparison, H. Mert et al. [26] used an unsaturated polyester, glycidyl methacrylate, and divinylbenzene to prepare micrometer-sized microspheres, which were functionalized with amine and used for metal removal. On the other hand, Cui et al. [27] synthesized styrene- and acrylate-based microspheres using suspension polymerization for oil spill clean-ups. The microspheres had an average size of 320 μm; however, the prepared microspheres had a closed porous structure on the surface and an interconnecting surface on the inside. In addition to the environmental applications, porous microspheres can be used for biomedical applications, which were demonstrated by Cosgriff-Hernandez et al. [28]. For the synthesis of ethylene glycol dimethacrylate (EGDMA)-based microspheres with tuneable diameters ranging from 200 to 800 μm, fluidics were used. The prepared microspheres achieved a high encapsulation efficiency (73%) of the growth factor rhBMP-2. In another study, the same group prepared two differently sized EGDMA-based microspheres (400 and 800 μm) with differently sized pores (15 and 30 μm) and loaded them with rh BMP2. These microspheres were then incorporated into an HIPE to form a microsphere-polyHIPE composite after curing [29]. Paterson et al. [21] demonstrated the use of acrylate-based microspheres for cell cultures by preparing them with either a continuous stirred tank reactor (CSTR) or a microfluidic device. 

From the mentioned methods, microfluidics seems to be the most suitable to prepare porous microspheres with a desired diameter and a uniform size. However, the main drawbacks of this method are its complexity and the amount of microspheres produced in a given time. In comparison, using traditional suspension polymerization, scale-up is possible. Nonetheless, not much research has been conducted on the preparation of polyHIPE microspheres using suspension polymerization, as emulsions tend to coalesce due to their instability. Additionally, most of the suspension polymerizations have been carried out using thermal initiation, which tends to further destabilize the emulsion and increase coalescence [30].

Considering all these shortcomings, multiple emulsions, thiol-ene chemistry, and photoinduced polymerization were combined to prepare polyHIPE microspheres with an open porous structure and yields above 90%. By using photopolymerization, the additional destabilization of thermally induced polymerization is avoided, as there is no heating that would destabilize the emulsion. Additionally, the polymerization begins at the surface, which prevents the microspheres from polymerizing together. Given that thiol-ene chemistry was used to prepare the microspheres, the effect of the thiol-to-alkene ratio on the properties of the materials was studied (morphology, degradation, and surface area). The functionalization capabilities of the prepared microspheres were also investigated as this would greatly increase their applicative possibilities.

## 2. Materials and Methods

### 2.1. Materials

Monomers pentaerythritol tetrakis(3-mercaptopropionate) (PETMP, >95%; Darmstadt, Germany, Sigma-Aldrich), trimethylolpropane tris(3-mercaptopropionate) (TMPTMP, ≥95%; Darmstadt, Germany, Sigma-Aldrich), and trimethylolpropane triacrylate (TMPTA; Paris, France, Sartomer (Arkema Group)); surfactants polyvinylpyrrolidone K 90 (PVP K90; Darmstadt, Germany, Sigma-Aldrich), and Hypermer B246 (HB246; Snaith, UK, Croda); initiator Irgacure 784 (I784; Ludwigshafen, Germany, BASF); calcium chloride hexahydrate (CaCl_2_ · 6H_2_O, 98%; Darmstadt, Germany, Sigma-Aldrich); sodium hydroxide (≥98%; Darmstadt, Germany, Sigma-Aldrich); 5,5′-dithiobis(2-nitrobenzoic acid) (Ellman’s Reagent, ≥98%; Darmstadt, Germany, Sigma-Aldrich); ethanol (Milano, Italy, Carlo Erba); tetrahydrofuran (THF; Milano, Italy, Carlo Erba); N,N-diisopropylethylamine (≥99%; Darmstadt, Germany, Sigma-Aldrich); and methylene blue (MB; Darmstadt, Germany, Sigma-Aldrich) were used as received.

### 2.2. Preparation of Porous PolyHIPE Microspheres

**Figure 1:**
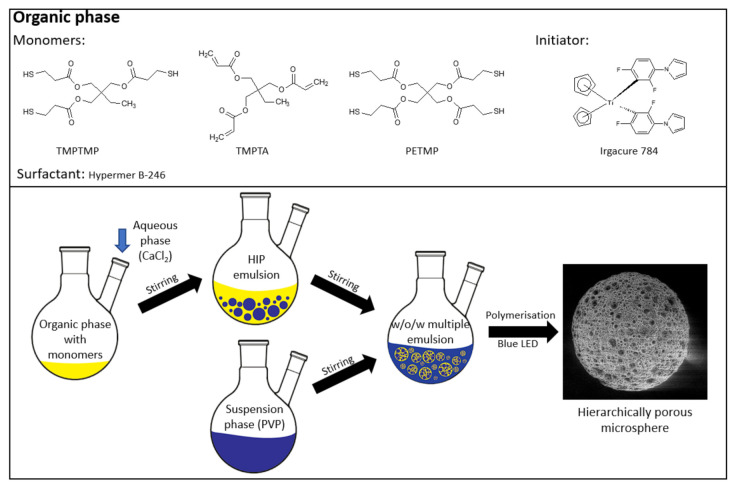
Microsphere preparation.

Highly porous microspheres were prepared from water-in-oil high internal phase emulsion (HIPE) precursors. High internal phase emulsions were prepared as follows: TMPTA (1.797 g, 6 mmol), PETMP (2.220 g, 4.5 mmol), or TMPTMP (2.410 g, 6 mmol); HB246 (2.5 wt %); I784 (2.5 wt %); and toluene (40 wt %) were added into an aluminum foil-covered 2-necked round bottom 250 mL reactor. The aqueous phase was prepared separately by dissolving calcium chloride hexahydrate (0.229 g) in deionized water (13 mL). The aqueous phase was added dropwise to the organic solution while stirring with an overhead stirrer at 350 rpm. Once the entire aqueous phase was added, stirring continued for another 20 minutes to produce a uniform w/o emulsion. An aqueous suspension phase consisting of polyvinylpyrrolidone K90 in 125 mL of deionized water was prepared separately. The aqueous suspension phase was added to the HIPE at a stirring speed of 400 rpm to obtain a stable w/o/w emulsion. The aluminum foil was removed once the entire suspension phase was added to the HIPE. The polymerization was carried out using a custom-made photoreactor consisting of five 1 W blue LED lights with a wavelength between 455 and 460 nm and irradiated for 2 h while stirring the multiple emulsion. The obtained particles were filtered and washed with water and acetone. The filtered particles were then continuously extracted with acetone in a Soxhlet apparatus for 24 h and dried under vacuum (for various compositions, see Table 1 and Table 2). A schematic representation of the entire process is shown in Figure 1.

The samples are labeled based on the thiol used (A for PETMP and B for TMPTMP), while the numbers represent the thiol to double-bond content (1 is 1:1; 2 is 1:2; 3 is 1:3).

### 2.3. Degradation Experiments

A measured mass of the microspheres was placed into a beaker, and a 0.1 M NaOH (aq) solution was added. Periodically, samples were washed in deionized water, left to air dry for 24 h, and dried in vacuum until constant mass. The percentage of mass loss was calculated:

(1) $$\%\;mass\;loss=\left(\frac{original\;mass\;–\;finals\;mass}{original\;mass}\times \;100\%\right)$$

### 2.4. Free Thiol Determination (Ellman’s Assay)

The available thiol groups were determined using a colorimetric assay described by Langford et al. [31]. Briefly, 25–50 mg of the microspheres was transferred into a 25 mL volumetric flask, and 5 mL of THF was added to the microspheres. The microspheres were left to swell for 30 minutes. Meanwhile, a 5 mL solution of Ellman’s reagent (25 μmol) in ethanol was prepared. The solution and 25 μL of N,N diisopropylethylamine were added to the microspheres. Afterward, the solution was diluted to 25 mL with ethanol and shaken for 30 min. Lastly, the absorbance of the mixture was measured at 412 nm.

### 2.5. Functionalization

Then, 200 mg of the polyHIPE microspheres was put into a two-necked flask and toluene was added to the microspheres. The microspheres were left to swell for 30 min. After the swelling, AIBN (5 wt %) and allylamine (100 times the measured value of the available thiol groups) were added. Subsequently, the reaction was carried out at 60 °C for 24 h while stirring under a nitrogen atmosphere. The functionalized microspheres were washed with toluene and ethanol, continuously extracted with ethanol in a Soxhlet apparatus for 24 h, and dried under vacuum.

### 2.6. Adsorption Experiments

The adsorption studies were conducted similarly to the study performed by Golub et al. [5]. Firstly, an aqueous solution of methylene blue (MB) with a concentration of 50 mg L^−1^ was prepared. Approximately 25 mg of dry beads was submerged in 15 mL of the dye solution and kept at room temperature. The samples were kept in the solution for a certain time, specified in Table 5. The concentration of the dye was measured using a UV–Vis spectrometer (λmax = 663 nm). The amount of adsorbed dye was calculated according to Equation (2):

(2) $$q_{t}=\frac{(c_{o}-c_{t})*V}{m}$$

where *c_o_* (mg L^−1^) is the initial dye concentration and *ct* (mg L^−1^) is the dye concentration at time t, *V* (L) is the volume, and *m* (g) is the mass of the dry samples.

### 2.7. Characterization

Particle size distribution was measured with an optical microscope with a digital camera (Novex; The Netherlands) by measuring the diameter of at least 100 microspheres. Elemental analysis was conducted using a PerkinElmer CHNS/O 2400 Series II analyzer. SEM images were taken on an FEI Sirion 400 NC scanning electron microscope, while the cavity and window sizes were measured from the SEM images using Image J software. UV–Vis measurements were conducted on an Agilent 8453 UV-Visible spectrophotometer.

## 3. Results and Discussion

**Figure 2:**
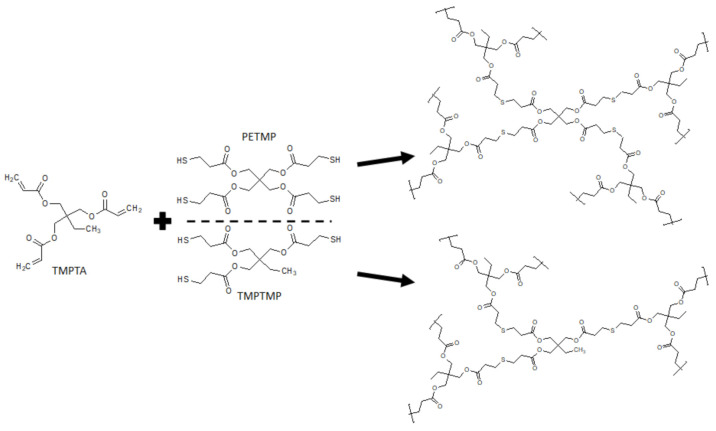
Polymerization scheme of the prepared trithiol- and tetrathiol-based microspheres.

To prepare highly porous polyHIPE microspheres in an efficient batch approach, a multiple emulsion system was used. This approach was already used [24]; however, the synthesis of microspheres with a fully open porous surface using this method tends to be challenging as many factors affect the results. Therefore, great emphasis was placed on the preparation of microspheres with a fully interconnected porous structure and open surface. Thiol-ene photopolymerization was chosen as the polymerization mechanism as it has been shown to be efficient for the preparation of porous monolithic polyHIPE materials [32,33]. The polymerization scheme of the synthesized microspheres is shown in Figure 2.

The highly porous microspheres were generated using multiple emulsions, which had a high internal phase emulsion as their primary phase and an aqueous solution of polyvinylpyrrolidone K90 as the secondary phase. The HIPE consisted of a thiol and an acrylate, which were stabilized with the polyoxyalkylene-modified block copolymer surfactant Hypermer B246 and had an internal phase volume share of 80%. The multiple emulsion was formed by adding the suspension phase to the HIPE. If, on the contrary, the HIPE was added to the suspension phase, it would already start to cure during the addition as it would be exposed to daylight, which would initiate the polymerization. Therefore, it was necessary to add the suspension phase to the HIPE to enable the successful formation of a w/o/w emulsion. Additionally, the use of an appropriate stabilizer, namely, polyvinylpyrrolidone K90, was also crucial to form a stable w/o/w emulsion. According to prior studies, it was observed that better emulsion stability was obtained when a higher molecular weight PVP was used.

**Figure 3:**
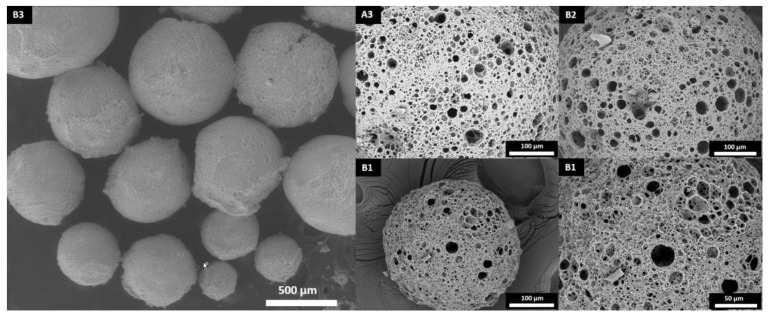
Surface morphology of the polyHIPE microspheres.

The experiments were carried out using a titanium-based photoinitiator (Irgacure I784) as its absorption peak is in the visible-light spectrum (high absorption of light is between 400 and 480 nm) [34,35]. A custom-made photoreactor was built using blue LED lights with wavelengths between 455 and 460 nm. The use of this photoreactor enabled the production of porous polyHIPE microspheres with an open-cell porosity (Figure 3) and yields above 93% (Table 3). Elemental analysis was conducted to measure the sulfur content, resulting in sulfur contents close to the calculated values (Table 3).

Thermally initiated polymerization was also attempted, as it is usually more accessible (cheaper initiators and sources of initiation). Using AIBN did not result in the successful synthesis of porous microspheres, and aggregated particles were formed. This was most likely due to the destabilization caused by heating, as observed in the ageing mechanism of perfluorocarbon emulsions [30].

In order to investigate the influence of the thiol-to-acrylate ratio and thiol functionality on the diameter, morphology, and degradation, particles with various thiol-to-acrylate group molar ratios (1:1, 1:2, and 1:3) and two thiols (TMPTMP and PETM) were prepared. Thiol-acrylate polymerization typically follows a mixed-mode polymerization mechanism, comprising of both step-growth and chain-growth polymerization. This leads to the thiol-ene polymerization of thiols and acrylates (step-growth) and the homopolymerization of acrylates (chain-growth) [36]. By varying the thiol-to-acrylate ratio in favor of acrylate, the polymerization favors homopolymerization. Consequently, both the thiol type and acrylate ratio affected the properties of the spherical particles. The diameters of the prepared microspheres were between 411 and 500 µm (Table 3, Figures S1 and S2). The acrylate content did not significantly affect the microsphere size; however, the PETMP-based microspheres were slightly larger as their diameters ranged from 439 to 500 µm, as opposed to the TMPTMP-based microspheres’ diameters (411 to 435 µm). The size dispersion of all prepared samples was, as expected, relatively high, which is typical for the suspension polymerization process as the reactor geometry dictates the centrifugal force at stirring, which influences the sphere size. 

**Figure 4:**
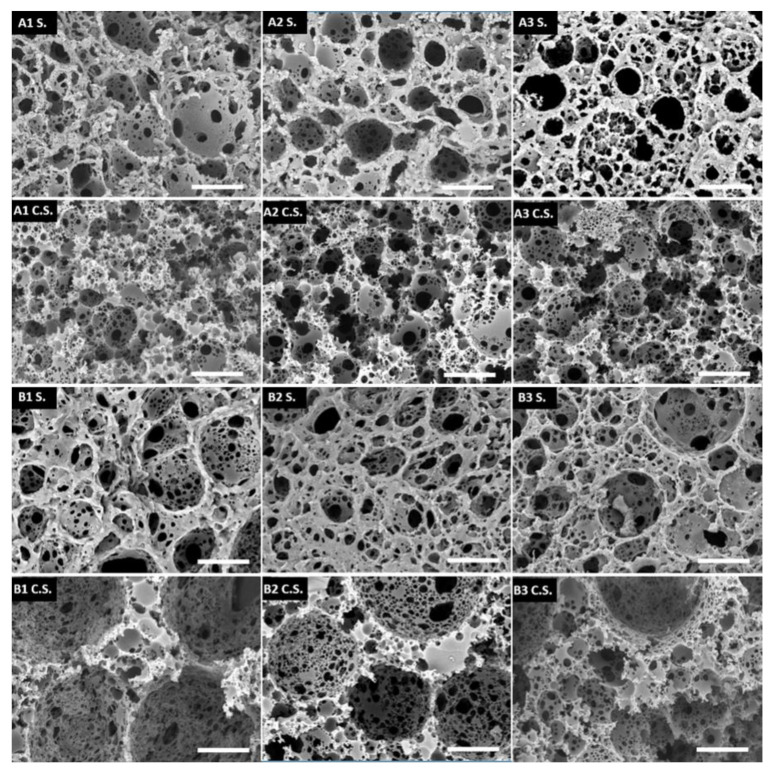
SEM pictures of the surface morphology (S) and the cross-section morphology (C.S.). The scale bar represents 20 μm.

It needs to be emphasized that all of the prepared microspheres had an open porous structure on both the surface and the inside, which is well-visible from the SEM images (Figure 4). The main difference between the surface and inner morphology is the amount of pores present on the surface and the interconnectivity, which is slightly lower on the surface. This could be due to the interactions between the suspension phase and the internal phase of the HIPE (both are composed of water), which affect the surface morphology. Although there was a slight difference in the microsphere diameter between trithiol and tetrathiol, interestingly, the cavity and window diameters of the microsphere surface were approximately the same. However, while the cavity diameter of the surface did not differ significantly, the cavity diameter of the cross-section was larger in the case of TMPTMP-based microspheres, especially in sample B1, which had cavities approximately three times larger than any of the PETMP-based samples (Table 4). This is most likely due to the difference in the HIP emulsion stability, which seems to be larger for tetrathiol. Additionally, by adding higher acrylate content, the cavity diameter of the cross-section in trithiol decreased from 30.0 to 16.1 (B2) and 13.4 μm (B3), indicating that the acrylate content influences the HIPE stability in the trithiol system and, therefore, the cavity diameter.

**Figure 5:**
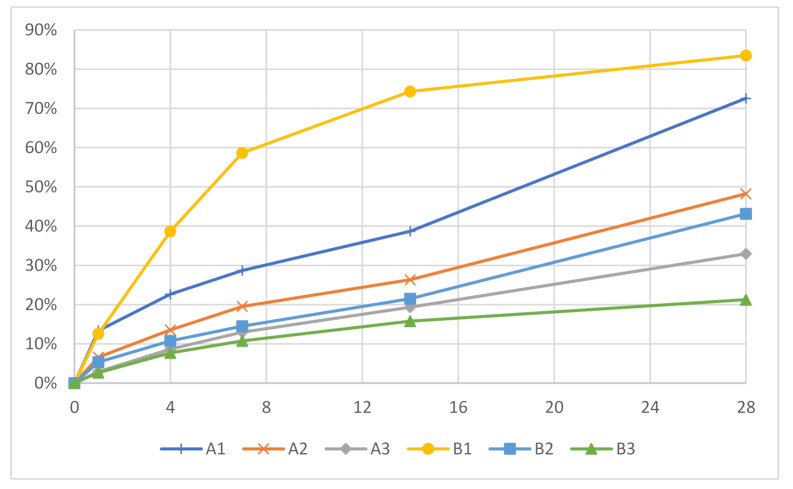
Degradation degrees of the polyHIPE microspheres.

In addition to the effect on the morphology, the acrylate content considerably influenced the degradation speed, as it decreased with increased acrylate content, which is shown in Table 5. Samples A1 and B1 had considerably higher degradation rates compared to the samples with higher acrylate content. A1 and B1 had degradation degrees of 72.6% and 83.5% at 28 days, respectively; while A2, A3, B2, and B3 had degradation degrees of 48.2%, 32.9%, 43.1% and 21.3%, respectively. The degradation of most samples increased steadily up to 28 days, with the exception of B1 (Figure 5). Sample B1 had a decrease in its degradation rate in the later stages, as it only increased by 9.1% between 14 and 28 days. This plateau-like effect is probably due to the fast degradation of the thioester bonds, which resulted in a slower degradation in the later stages as fewer degradable bonds were left at the end. It was demonstrated that by varying the acrylate content, it is possible to control the degradation degree. This was also shown by Rydholm et al. [36].

The availability of free thiol groups was evaluated using Ellman’s assay, as free thiol groups would be used for further functionalizations of the material. Sample B1 had 74.04 μmol/g of available thiol groups, whereas B3 had 19.98 μmol/g of available thiol groups. This result was expected as sample B3 had significantly more acrylate content (3:1) compared to sample B1 (1:1). Therefore, more of the thiol groups were used up during the thiol-ene polymerization. Subsequently, samples B1 and B3 were functionalized with allylamine using free thiol groups and a radical source in order to demonstrate the availability of polyHIPE microspheres for chemical functionalization. After the functionalization and purification, the functionalized microspheres had a brown color, indicating the successful functionalization with allylamine (Figure S3). The functionalization was monitored by the determination of free thiol groups and the elemental analysis. The amount of free thiol groups in sample B1 reduced from 74.04 to 22.34 μmol/g, while the amount of free thiol groups in sample B3 reduced from 19.98 to 7.88 μmol/g. Additionally, the elemental analysis confirmed the presence of nitrogen in both samples (1.87% in B1 and 1.00% in B3). However, since the nitrogen content was higher than the theoretically calculated values, this suggests that allylamine can also bind to the unreacted acrylate double bonds. 

To further demonstrate the applicative possibilities of the polyHIPE microspheres, they were used in the adsorption of methylene blue. Methylene blue is regularly used as a dye in the textile industry, which results in the toxic pollution of wastewater [37]. Additionally, methylene blue was shown to have a neurotoxic effect on the central nervous system [38]. The B1 microspheres were shown to adsorb over 40% (Table 6) of the MB present in the MB solution. Intense coloration of the microspheres was observed (Figure S3). The microspheres differed in their maximum adsorption capacity, which was 12.0 mg/g for B1 and 7.8 mg/g for B3. Additionally, the B3 microspheres reached their maximum adsorption capacity after 120 min. In comparison, the B1 microspheres adsorbed 6.7 mg/g of the dye in 2 h as opposed to their maximum capacity of 12.0 mg/g.

## 4. Conclusions

A facile method for the preparation of hierarchically porous microspheres with interconnected cellular morphology within a batch process was demonstrated. This method results in considerably higher efficacies compared to other synthetical methods. The use of visible-light photopolymerization and the thiol-ene click reaction enabled the preparation of microspheres with open surface porosity and tuneable degradability. The multiple emulsion approach allows for easy scale-up while thiol-ene chemistry adds the possibilities of many commercially available monomers. The highly porous interconnected morphology was shown to be beneficial for chemical functionalization and dye adsorption, while the high pore volume of the novel microspheres allows for the capability of adsorption and absorption processes, leading to numerous possible applications.

## Figures and Tables

**Table 1:** Preparation of PETMP-co-TMPTA polyHIPE microspheres.

Sample	m(TMPTA) (g)	m(PETMP) (g)	m(HB246) (g)	m(toluene) (g)	Aq. Ph. (%)	m(I784) (g)	thiol:ene
A1	1.797	2.220	0.101	2.394	80	0.102	1:1
A2	2.402	1.500	0.102	2.414	80	0.097	1:2
A3	2.698	1.140	0.101	2.421	80	0.098	1:3

**Table 2:** Preparation of TMPTMP-co-TMPTA polyHIPE microspheres.

Sample	m(TMPTA) (g)	m(TMPTMP) (g)	m(HB246) (g)	m(toluene) (g)	Aq. Ph. (%)	m(I784) (g)	thiol:ene
B1	1.798	2.410	0.105	2.606	80	0.100	1:1
B2	2.378	1.602	0.101	2.546	80	0.102	1:2
B3	2.690	1.204	0.099	2.478	80	0.096	1:3

**Table 3:** Microsphere size, calculated and measured sulfur content, and yield.

Sample	Average Microsphere Diameter (µm)	Found S (%)	Calculated S (%)	Yield (%)
A1	439 ± 157	12.80	14.51	95
A2	491 ± 157	10.06	9.56	95
A3	500 ± 162	7.76	8.01	97
B1	411 ± 129	12.61	13.82	93
B2	435 ± 154	9.53	9.69	94
B3	425 ± 133	7.41	7.47	97

**Table 4:** Cavity and window diameter of the surface and cross-section of trithiol- and tetrathiol-based microspheres.

Sample	Surface Cavities (µm)	Surface Windows (µm)	C.S. Cavities (µm)	C.S. Windows (µm)
A1	9.4 ± 3.8	1.3 ± 1.1	9.7 ± 4.2	1.2 ± 0.6
A2	9.6 ± 4.3	1.3 ± 0.9	8.9 ± 3.9	1.4 ± 0.9
A3	13.2 ± 5.7	1.9 ± 1.4	10.1 ± 4.9	1.5 ± 1.1
B1	10.6 ± 5.3	1.9 ± 1.1	30.0 ± 8.7	1.7 ± 1.0
B2	14.1 ± 7.0	2.4 ± 1.4	16.1 ± 11.0	1.8 ± 1.3
B3	15.5 ± 9.0	2.1 ± 1.5	13.4 ± 9.2	1.5 ± 0.8

**Table 5:** Degradation degree of the produced porous microspheres in 0.1 M NaOH.

0.1 M NaOH Degradation					
Sample	1 Day (%)	4 Days (%)	7 Days (%)	14 Days (%)	28 Days (%)
A1	13.3	22.7	28.7	38.7	72.6
A2	6.6	13.6	19.5	26.4	48.2
A3	2.9	8.6	13.0	19.3	32.9
B1	12.6	38.7	58.7	74.4	83.5
B2	5.4	10.7	14.5	21.5	43.1
B3	2.7	7.7	10.8	15.8	21.3

**Table 6:** Amount of adsorbed methylene blue at specified time points.

	Methylene Blue Adsorption			
Time (min)	q_t_ (B1; mg/g)	q_t_ (B3; mg/g)	B1 (MB Removed; %)	B3 (MB Removed; %)
15	1.8	2.6	6.8	8.6
30	3.3	4.1	11.3	14.0
60	3.7	6.0	12.8	21.5
120	6.7	7.8	23.0	26.9
1440	12.0	7.8	40.8	27.7

## Supplementary Materials

The following are available online at https://www.mdpi.com/article/10.3390/polym13193366/s1, Figure S1: Size distribution of the tetrathiol-based microspheres, Figure S2: Size distribution of the trithiol-based microspheres, Figure S3: Microspheres appearance after functionalization and adsorption.
