# Supplementary material for: Hierarchically Porous Microspheres by Thiol-ene Photopolymerization of High Internal Phase Emulsions-in-Water Colloidal Systems
Source: Polymers (Basel). 2021 Sep 30;13(19):3366. doi: 10.3390/polym13193366 (PMC8512400; doi:10.3390/polym13193366)
Supplement: Supplementary file 1 [file polymers-13-03366-s001.zip › polymers-1399491-supplementary.pdf]

polymers

Supplementary Information

Hierarchically porous microspheres by thiol-ene photopolymerization of high internal phase emulsions-in-water colloidal systems

Stanko Kramer, Peter Krajnc*

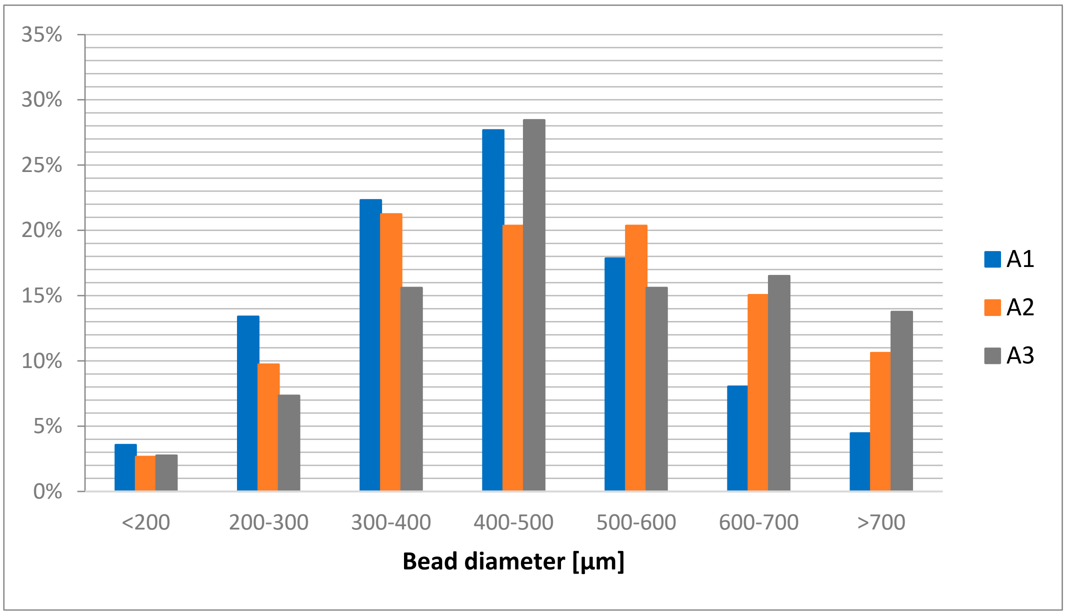

Figure S1. Size distribution of the tetrathiol based microspheres.

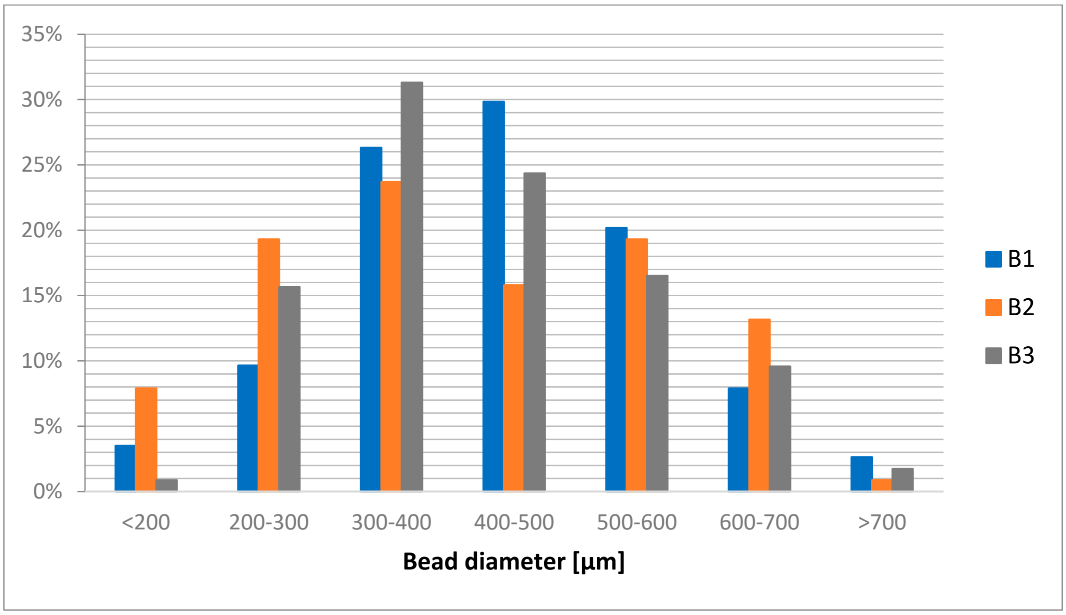

Figure S2. Size distribution of the trithiol based microspheres

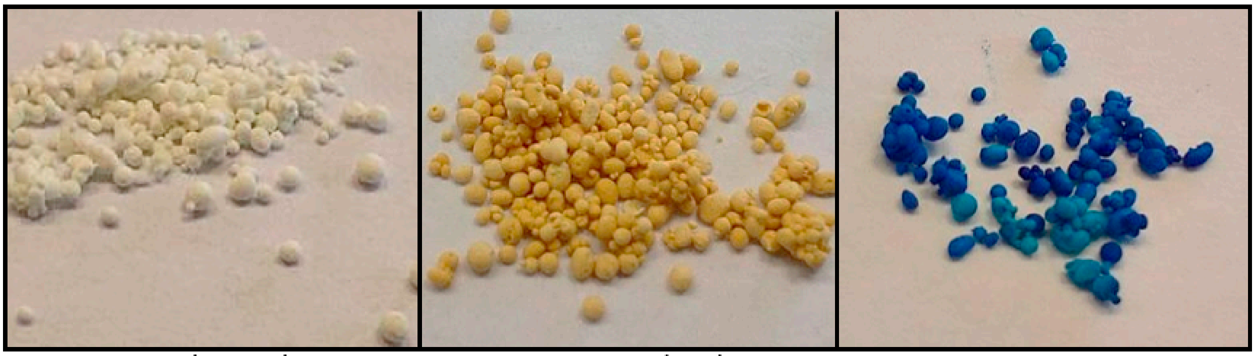

Figure S3. Microspheres appearance after functionalizatioon and adsorption
